# Construction and simulation of a joint scale model for power electronic converters based on wavelet decomposition and reconstruction algorithms

**DOI:** 10.1371/journal.pone.0298590

**Published:** 2024-04-05

**Authors:** Jianhua He

**Affiliations:** School of Transportation Engineering, Jiangsu Vocational Institute of Architectural Technology, Xuzhou, China; Wroclaw University of Science and Technology: Politechnika Wroclawska, POLAND

## Abstract

In power electronics systems, system design and operation often involve multiple time and space scales, ranging from nanosecond switching dynamics to hour-level system operation behavior. Due to the complexity of these systems and the rise of wide-gap semiconductor technology, a series of multi-scale phenomena have emerged that are difficult to ignore. The high frequency of switching operations makes multi-scale effects particularly significant, including the fast dynamic response of the power loop, EMI, and heat conduction problems. They are key factors that must be considered in the design to ensure the efficient and reliable operation of power electronic devices. This study proposes the construction and simulation of a joint scale model for power electronic converters based on wavelet decomposition and reconstruction algorithms to address the multi-scale phenomenon and limitations of single-scale power electronic converters. Firstly, a joint scale model for power electronic converters at both macro and micro-scales was established, targeting both single-scale models and simple combinations of multiple scale models for power electronic converters. The traditional single-scale model is sufficient to describe the average behavior of the converter, but it has serious limitations in capturing fast transient processes and high-frequency switching behavior in power electronic systems. These limitations often manifest themselves when there is a need to capture fine timescales of detail. By transforming between the time domain and the frequency domain, wavelet decomposition enables the model to capture both macroscopic average characteristics and microscopic transient dynamics. The wavelet reconstruction algorithm can simulate all kinds of fast changes in the actual working process more accurately and compress irrelevant information while retaining key signal features, so as to optimize the simulation performance of the model. Secondly, this algorithm is used to analyze BC in short time scale. Finally, the short time scale characteristics of power electronic converters are analyzed. Experimental results show that the fusion of wavelet decomposition and reconstruction algorithm enhances the accuracy of the power electronic converter model and improves the performance of the system. The model achieves an error reduction of nearly 3% in the calculation step size of 10^-7^s, which has a significant impact on the high precision requirements of high-frequency operations. In addition, the optimal calculation step size of 8×10^-8^s achieves an error reduction of more than 14%, making an important contribution to the transient analysis and fine structure simulation. The wavelet algorithm can improve the accuracy of multi-scale modeling in power electronic system and reduce the simulation time. The reduction of error not only shows the improvement of the accuracy of the model, but also shows its practical significance in the design and test of the actual power electronic system. The reduction in error reveals the ability to more accurately predict and mitigate potential performance problems in matching tests with actual hardware, as well as its ability to adapt to emerging wide bandgap semiconductor materials and structures.

## 1. Introduction

Wavelet Transform (WT) is widely applied when processing signal, which is often applied to signal singularity detection, short-term signal recovery, and non-stationary signal denoising [1]. Since it was proposed in the 1980s, WT has become a powerful time-frequency analysis tool. It can reveal the local characteristics of signals at different scales, and is especially suitable for the analysis of non-stationary signals with complex frequency behavior [2]. Due to its unique multi-resolution analysis capability, it has also been widely used in medical imaging, geological exploration, and financial market analysis [3]. This method decomposes the signal into a series of sub-band wavelets, each with a different representation [4]. Therefore, before WT, the signal needs to be decomposed and reconstructed [5]. The multi-resolution characteristics of WT make it an ideal tool to identify and analyze abrupt and atypical features in signals [6]. For example, in power line monitoring, WT can help engineers identify and locate faults, thereby reducing detection time and improving response speed [7]. With the rapid development of information technology and the increasing demand for electricity, Power Electronic Converter (PEC) is very important in providing efficient and reliable electrical energy conversion. It is difficult for traditional single-scale models to accurately capture abrupt changes and nonlinear features in a short time [8]. PEC plays an important role in modern power electronic devices and has become an important direction in the development of power electronic technology [9]. PEC is an indispensable part of the modern power system. The converter plays a crucial role in the integration of renewable energy sources such as solar power and wind power, as well as in battery management systems for electric vehicles. Modern applications such as charging devices for electric vehicles and solar power inverters demonstrate the key role of PEC in enabling green energy conversion and improving energy efficiency. To solve the shortcomings of existing models in describing the comprehensive behavior of PEC, a joint scale model of PEC based on multi-scale considerations of time and space is constructed. By using Wavelet Decomposition (WD) and Reconstruction Algorithm (RA), not only the detail capturing power of the model is improved in short time scale, but also the overall simulation accuracy is enhanced. The contribution of the research is to the possibility of practical engineering applications, improving the design reliability and operational performance of PEC in the power industry where technology is rapidly evolving to meet the growing high standards of the market. However, although PEC has been extensively studied, most of the models presented in the existing literature are still based on a single-scale. This limitation leads to deficiencies in simulating rapid changes and handling multi-physical interactions, which in turn prevents more accurate system performance prediction and optimization. The main contribution of this research is to the emerging smart grid and other related technologies, such as advanced battery systems for energy storage and management. They have potential applications and drive the development of power systems in a more economical, efficient and reliable direction. The research contents mainly include five parts. Firstly, the introduction describes PEC failure at a short time scale in the contemporary context of information technology development. Then there is a literature review on the applications of WD and RA in various fields, as well as the current research status of many scholars on these two technologies. In the third part, an effective joint scale model is established to obtain accurate models at various single-scales and the coupling relationship between these different scale models, and the simulation calculations of the model are studied. The first section analyzes the joint scale model under WD and RA. The second section studies the joint scale modeling of Buck Converter (BC). The third section analyzes the joint scale model and short-term scale characteristics of Dual Active Bridge (DAB) converter. In the fourth part, through comparative experimental analysis, the accuracy and effectiveness of the algorithm for the PEC multi-scale model, as well as the resolution of conflicts in simulation calculations caused by scale issues, are demonstrated. Finally, there is a summary and outlook on the research methods and results of this study. Through the study of WT technology, the characteristics of each model can be calculated more accurately. And it can better meet the needs of the vast number of users on the safety and electrical characteristics of PEC. Meanwhile, it can accurately and effectively solve the contradiction of scale problems in the simulation calculation.

## 2. Related works

WT is a method of decomposing signals into sub-band signals of different scales and frequencies and analyzing them to extract useful information. WT is widely applied, including image processing, voice signal processing, remote sensing data analysis, and medical imaging analysis, and many scholars have related research. Rezvani H and Khodadi H proposed a method based on evolutionary wavelet neural networks. Power quality index was used to estimate the slag quality. They applied evolutionary methods to train the parameters and wavelet combinations of the model. All electrical parameters of the smelting process are measured using an energy quality analyzer to evaluate the quality of the foaming slag. The highest accuracy can be obtained by using five leading indicators. The accuracy of this method in estimating foam slag quality is 99%, which shows the effectiveness of this method [10]. Li J et al. proposed a new statistical model, which contained multiple uncertainties. This model was used to capture local nonlinear changes in joint probability distribution. The model contained multiple uncertainties within a random Galerkin framework. The multidimensional tensor product of Haar wavelet expansion was used to capture local nonlinear changes in the joint probability distribution, and an operator-splitting-based approach was used to ensure that the modeled system remains hyperbolic. Joint uncertainty was introduced to match the evolution of quicksand beds driven by sudden dam failures and landslide dam failures, as well as the large-scale rapid evolution of quicksand beds in response to flash floods. This can be used to quantify various uncertainties in practical shallow water hydrological sediment morphology dynamics modeling applications [11]. Yehia A et al. proposed a fusion method based on WT and Intensity Hue Saturation (IHS) transform, which injected Synthetic Aperture Radar (SAR) data into optical images. A fusion method based on WT and IHS transform was used to inject SAR data into optical images. Both the spectral information of the original image and the spatial content of the high-resolution SAR image were preserved. Different fusion outputs were compared using different image quality indices. The visual and statistical results of the fusion output indicate that this method has effective conversion from SAR to optical images, enhancing the interpretability of SAR images [12]. Dinklage P et al. proposed a sequential and parallel algorithm for constructing wavelet trees, which refined the characters represented in nodes of tree in the opposite way as depth increases. First, a new sequential algorithm in RAM and external memory was described. Then these algorithms were applied to parallel computers dealing with shared memory and distributed memory settings. The experimental results show that the algorithm is superior to previous algorithms in terms of time and memory efficiency and can compute all auxiliary information based on the information obtained from computing leaves. The algorithm is also suitable for wavelet matrices and is suitable for large alphabet variants. Although the algorithm works well with wavelet matrices and is particularly suitable for large alphabet variants, its computational complexity can increase with the amount of data, which may limit its application when dealing with extremely large data sets [13]. Malmir I proposed a new method based on quadratic programming wavelet. By direct derivation of Legendre and Chebyshev wavelets of arbitrary scale, the Caputo derivative matrix of Legendre and Chebyshev wavelets of arbitrary scale was introduced. The Caputo derivative operation matrix was used for quadratic optimization of systems with fractional or integer differential equations. By directly deriving the derivative operation matrices of arbitrary scale Legendre and Chebyshev wavelets, their properties were introduced. The derivative operation matrix was used for quadratic optimization [14].

RA is closely related to optimization algorithms. And many optimization problems have solutions that are the special cases of RA, such as the steepest descent method. Many scholars have applied this algorithm to multiple fields. Chen Z et al. proposed an ab initio algorithm for reconstructing the topological structure of glycans from tandem mass spectrometry, which included two main improvements of Xingtang biological GlycoNovo. The precursor mass measured using glycan peak was used to determine its potential components. And a program was developed to calculate reconstructed topology candidates’ empirical *p*-value. The experimental results prove the effectiveness of the proposed method. However, due to the high complexity of the algorithm, it may require large computational resources, so it may be difficult to apply in the environment with limited resources [15]. He L explored the application of 50% iterative RA in color Doppler imaging for diagnosing motor abdominal pain, as well as the changes in liver, gallbladder, and portal vein before and after exercise. Color Doppler blood flow imaging was used for examination, and iterative reconstruction with a weight of 50% was used for image reconstruction. These reconstructed images were subjectively and objectively evaluated. The signal-to-noise ratio after ultrasound image reconstruction was significantly improved compared to the value before ultrasound image reconst ruction. The results show that the signal-to-noise ratio after ultrasonic image reconstruction is significantly higher than that before ultrasonic image reconstruction. However, this method may have a certain impact on the details of the image, resulting in the loss of some details of the image. The minimum lethal dose of subjects in the 12th minute during exercise was significantly less than that in the 0th minute. However, the minimum lethal dose of subjects at 12th minute after exercise was significantly greater than that at 0th minute, reducing artifacts and noise interference [16]. Zhang Y et al. proposed a RA using an improved two-step transformation in interleaved SAR mode. To provide the prior knowledge required for reconstruction, that is, the non-uniform sampling distribution dependent on PRI changes, an optimal PRI change scheme is first designed and the sequence parameters are optimized. Using the designed PRIs sequence, non-uniform distance curvature correction and data reconstruction are realized simultaneously. This algorithm could jointly achieve non-uniform distance curvature correction and data reconstruction. This algorithm has significantly improved compared to traditional reconstruction methods in terms of peak sidelobe ratio, integrated sidelobe ratio, and azimuth ambiguity signal ratio, verifying the method effectiveness [17]. Zappa E and Marchisotti D proposed a program to obtain virtual datasets from any real 3D dataset collection, while fully controlling the environment, 3D sensors, and trajectory conditions. These datasets could be generated to test the effectiveness of algorithm parameters to determine the optimal parameters for utilizing algorithm characteristics. The best parameters are determined to leverage the characteristics of the algorithm for the best results in each operating environment to perfectly control each condition and assess its impact on the final result. Two RAs are used as examples, namely Open3D reconstruction tool and ElasticFusion. It could perfectly control each situation and evaluate its impact on the final result. The results show that the setting of the algorithm parameters can have a strong influence on the obtained 3D reconstruction and trajectory. However, because the parameters need to be fine-tuned to get the desired result, this can make the algorithm more difficult to use. The setting of algorithm parameters could have a strong impact on the obtained 3D reconstruction and trajectory. The impact of unclosed loop trajectories on reconstruction performance was quantified in experiments for different application scenarios [18].

In summary, current WD and RA can solve complex multi-scale problems in many fields, including power electronics. From high-precision slag quality estimation of electric arc furnaces to the application of uncertainty models in environmental engineering, to the improvement of image data fusion technology, WD methods are constantly optimized and applied to solve practical problems. In addition, RA shows considerable efficiency and accuracy in sequence analysis, image reconstruction, and 3D modeling. By referring to the characteristics of multi-scale analysis of WT and the successful application of RA in data quality optimization, a new path of joint scale model of PEC can be conceived. WD and RA solve various multi-scale problems in most fields, so they are applied to multi-scale problems in PEC. WD and RA are integrated into PEC and a joint scale model is constructed for PEC. It is hoped to obtain coupling relationships between different scale models and improve joint scale models’ accuracy and effectiveness. Although this can obtain the coupling relationship between different scale models and improve the accuracy and effectiveness of the joint scale model, it may also increase its complexity, which may have a certain impact on its computational efficiency. To address the conflicts in simulation calculations caused by scale issues, a PEC joint scale model construction and simulation experiments based on WD and RA are proposed.

## 3. Construction of pec joint scale model based on wd and ra

In PEC modeling, the mathematical model of converter is a foundation for establishing its accurate simulation model, and multi-scale analysis method is currently commonly used in PEC mathematical model research. Although there are many small signal models and simulators that can predict the power level conversion function and compensate accordingly, most of these models and methods use a single-scale model or simply combine multiple scale models. So it is difficult to accurately describe the complex characteristics of PEC under different conditions, especially under short time scales and unstable conditions. Due to the different manifestations of PEC under high-frequency small signals and low-frequency large signals, a joint scale model of PEC was constructed using WD and RA. This model is based on multi-scale analysis and obtains a joint scale model by decomposing and reconstructing the mathematical model of the converter, to achieve high simulation accuracy. The performance difference of PEC at different frequencies has always been a problem to be solved. To meet this challenge, this study provides a more efficient and accurate modeling method. Therefore, the proposed joint scale model is not only an improvement of the existing methods, but also a response to the current technical situation and challenges. It brings new possibilities for the simulation and research of PEC.

### 3.1. Joint scale model under wd and ra

**Fig 1:**
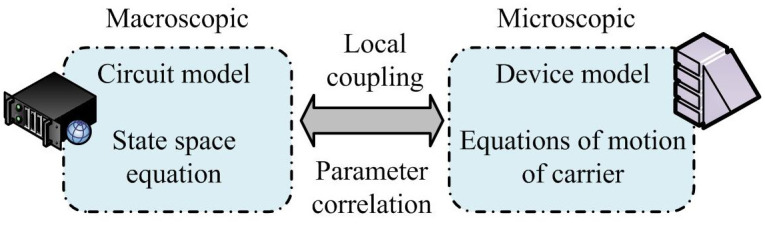
Coupling relationship between macro and micro-scale models in power electronic converters.

In the PEC model, the circuit model established based on circuit principles is divided into macro-scale models, and the PEC circuit model with high accuracy is a state space equation teaching model [19]. WT is an effective time-frequency analysis tool, which can subdivide the signal into different sub-band wavelets, and each sub-band can capture the characteristics of the signal at different scales or high- and low-frequency bands [20]. The necessity of signal reconstruction is that after WD, the signal can be analyzed more deeply in the time domain or the frequency domain. Then a more comprehensive and accurate signal model can be reconstructed by using these sub-band information. This decomposition and reconstruction process is particularly important when dealing with signals in PEC. And it can better represent the different performance of PEC in high-frequency small signals and low-frequency large signals. In typical PEC macro-scale models and macro-scale device models, there is a local coupling relationship that is interrelated. Fig 1 shows the coupling relationship between the macro and micro-scale models in PEC and these two.

There is a correlation between common and partial parameters in both models. The mathematical model *f*_*C*_(*u*_*i*_, *i*_*i*_, *r*, *L*, *C*) of PEC circuit was established using spatial equations. The mathematical model *f*_*D*_(Q*, *u*_*i*_, *i*_*T*_, *u*_*T*_) of power electronic devices is established using the carrier motion method. Among them, *u*_*i*_ is the input voltage of circuit, which is the most common parameter shared between two models. *ir* is the input current of switching device. *u*_*T*_ is the terminal voltage of switching device. When the switching device is connected to converter circuit, there is a certain mathematical relationship between *i*_*T*_ and the unique component parameters *r*, *L*, and *C* in the circuit, as well as between *u*_*i*_ and *u*_*T*_. So Eq (1) is an expression for *i*_*T*_.


$$i_{T}=f(u_{i},u_{T},r,L,C)$$ (1)

**Fig 2:**
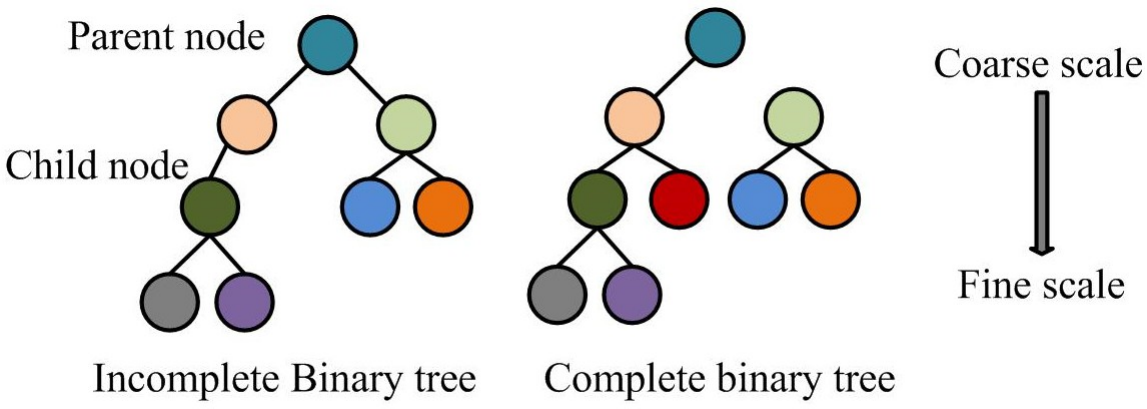
Homomorphic binary tree.

Eq (1) represents the parameter correlation coupling relationship between macro-scale circuit models and micro-scale device models in PEC. By connecting macro and micro-scale models, a joint scale model of PEC can be obtained. When WT and RA are separated from the multi-scale framework, the coarse and fine scale transformation in coarse and fine scale transformation principle of this combination algorithm is defined in homomorphic binary tree. Fig 2 shows its structure.

Homomorphic binary tree is a graph composed of node connections, which has no direction, is infinite, and does not cycle. Among them, *k* represents tree node. $k\tilde{\gamma }$ represents the parent node of *k*. This framework has two smoothing algorithm parts: fine-scale to coarse-scale filtering and coarse-scale to fine-scale filtering. The decomposition process is consistent with the concept of obtaining macro variable *S* through the compression operator *D* for micro variable *s*. The reconstruction process is consistent with the concept of obtaining *s* from *S* through the reduction operator *R*. According to the scale recursive dynamic model of the homomorphic Binary tree, for *x*(*k*), Eq (2) is a coarse scale to fine scale process.


x(k)=M(k)x(kγ)+N(k)w(k) (2)

In Eq (2), *w*(*k*) represents the white noise. *M*(*k*)*x*(*ky*) is a predicted value from coarse scale to fine scale. *N*(*k*)*w*(*k*) refers to a higher resolution information added during the transforming coarse scale to the fine scale. On a certain scale *i*, the dynamic processing *x*(*i*,*k*) can be transformed through the pulse response *n*(*k*) of low-pass filter to obtain smoother processing on a coarser scale. The calculation process is reversed, and *x*(*i*,*k*) achieves complete reconstruction by transforming *x*(*i* − 1, *k*) and *x*(*i* − 1, *k*) in Eq (3).


x(i,k)=∑n(k)x(i−1,k)+∑m(k)x(i+1,k) (3)

By using WD and RA, the switching process *S*_*J*_ of the joint scale model in Eq (4) can be obtained.


$$S_{J}=\sum h_{1}(t)S_{w}+\sum h_{2}(t)s_{w}$$ (4)

**Fig 3:**
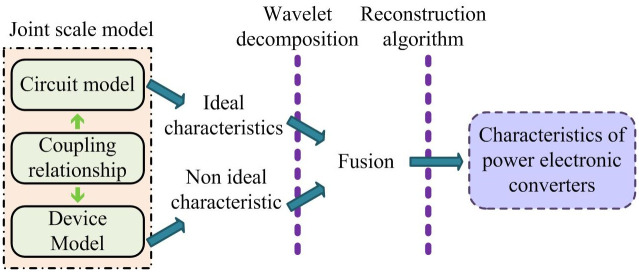
Schematic diagram of joint scale model and simulation for power electronic converters.

In Eq (4), *S*_*w*_ represents the macro-scale representation of switching after WD. *s*_*w*_ represents the micro-scale representation of switching after WD. By analyzing the coupling relationship between macro and micro-scales in PEC joint scale model, a schematic diagram of the PEC joint scale model and simulation is established in Fig 3. And it is combined with WD and RA of scale model, and the selection principle of unified calculation step size under this model.

PEC joint scale modeling and simulation method are to establish an accurate model of micro-scale circuit of PEC and an accurate model of micro-scale switch device. A joint scale model of PEC is established based on the analysis of duration and coupling relationship of switch characteristic processes in micro-scale models using a single-scale precise model. By combining WD and RA, a complete PEC joint scale simulation calculation model has been established.

### 3.2. Joint scale modeling of bc

**Fig 4:**
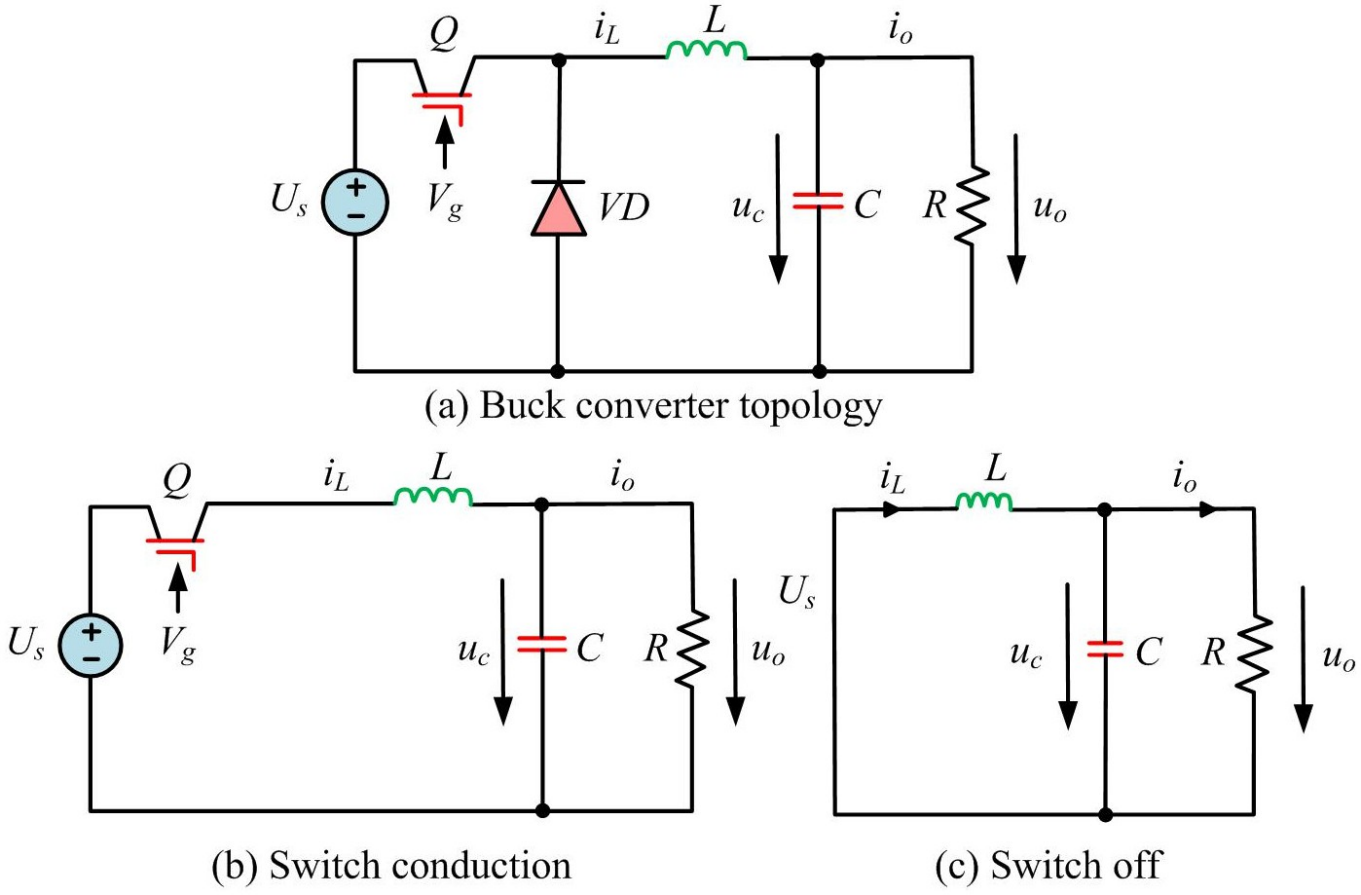
The two circuit states of a BC during continuous conduction and the topology of the converter. (a) Buck converter topology, (b) Switch conduction, (c) Switch off.

BC is a typical single switch DC-DC converter. The commonly used methods for establishing macroscopic circuit models of PEC include time average equivalent circuit method, state space average method, and three terminal switching device method [21]. For example, in the energy recovery system of electric vehicles, BC is used to convert the high voltage of the power battery to the low voltage suitable for on-board appliances. This process requires the converter to respond quickly to load changes and maintain a stable output as the battery is charged and discharged. Due to high-frequency switching operations, the handling of Electromagnetic Interference (EMI) and switching losses becomes a key challenge. In simulation analysis, the state space equation can more easily and accurately obtain circuit characteristics. Therefore, this study uses state space equation to construct a macroscopic scale model of BC circuit. Fig 4 shows two circuit states and topology of BC during continuous conduction.

For the Continuous Conduction Mode (CCM) of the BC during operation, the circuit can be divided into two modes based on the different states of switch Q. Among them, inductance current *i*_*L*_ and capacitance voltage *u*_*C*_ are used as state variables.

When Q is turned on, the freewheeling diode VD reversely cuts off, and Eq (5) is its state space equation.


$$\overset{\cdot }{x}=A_{1}x+B_{1}U_{s}$$ (5)

In Eq (5), *U*_*s*_ represents the input DC voltage. When Q is off, the continuous current diode VD is on. Eq (6) is its state space equation.


$$\overset{\cdot }{x}=A_{2}x+B_{2}U_{s}$$ (6)

Eqs (6) and (5) constitute the macroscopic circuit model of BC. Eq (7) is the expression of terminal voltage *u*_*ce*_ of switch Q within period *T*.


$$u_{ce}(t,0)=\left\{\begin{array}{ll} 0 & 0<t\leq DT\\ U_{s} & DT<t\leq T \end{array}\right.$$ (7)

Due to the high complexity of switching process, most studies use segmented fitting or linearization to describe it. The micro-switch device model takes Insulate-Gate Bipolar Transistor (IGBT) terminal voltage *u*_*ce*_ and the total internal residual carriers Q* as state variables. Eq (8) is the state space equation for its switching process.


$$\frac{dQ}{dt}=-\frac{Q}{\tau_{P}}-\frac{4Q^{2}I_{sne}}{W^{2}A^{2}q^{*2}n_{i}^{2}}$$ (8)

In Eq (8), *τ*_*p*_ represents the minority carrier lifetime in the IGBT base region. *I*_*sne*_ represents IGBT emitter electrons’ saturation current. *A* represents the total conductive area of IGBT. q* represents elementary charge. *n*_*i*_ represents the inherent carrier concentration in IGBT. There is a significant difference in terminal voltage’ numerical values of switch Q when switching transient at the macro circuit scale and micro device scale. IGBT switching is a process with multi-scale properties [22]. Based on WD and RA, Eq (9) represents the terminal voltage *u*_*ce*_ of Q.


$$u_{ce}(t)=\sum h_{1}(t)u_{ce}(t,0)+\sum h_{2}(t)u_{ce}(t,1)$$ (9)

**Fig 5:**
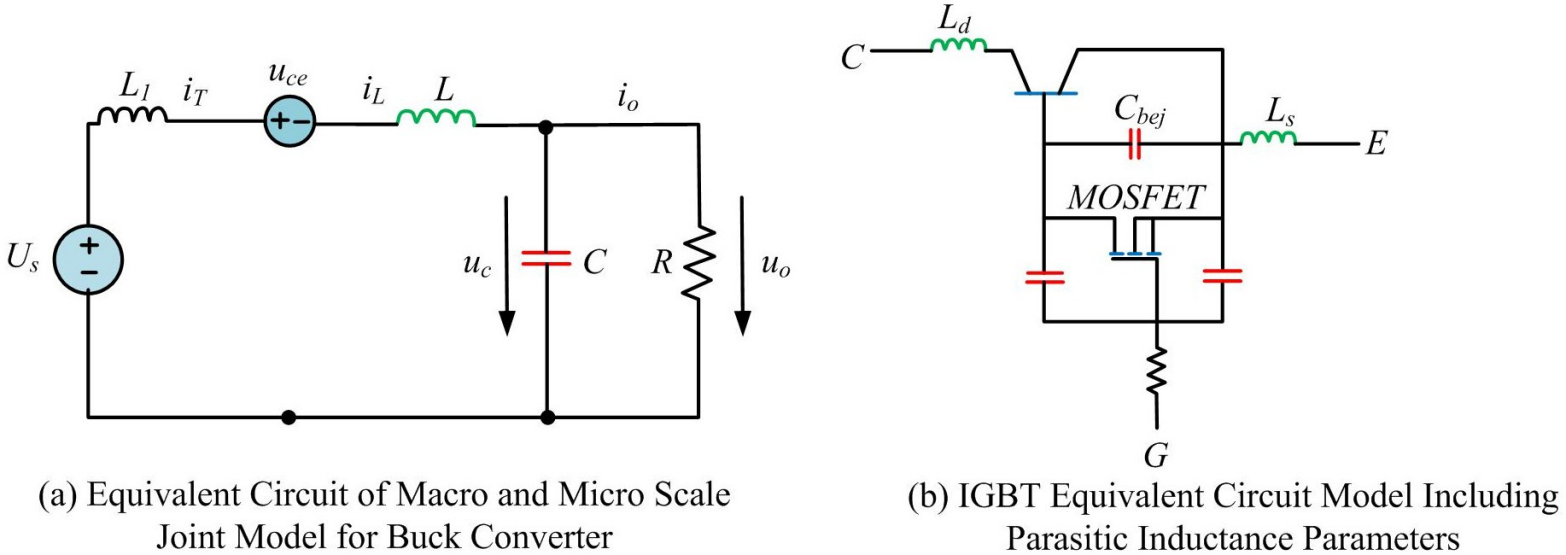
Equivalent circuit in buck converter circuit. (a) Equivalent Circuit of Macro and Micro Scale Joint Model for Buck Converter, (b) IGBT Equivalent Circuit Model Including Parasitic Inductance Parameters.

In Eq (9), *h*_1_(*t*) and *h*_2_(*t*) represent the orthogonal basis functions of WD. After transformation, the values of terminal voltage at different scales are fused, increasing the operational feasibility of their participation in calculations. In solving the equations in joint simulation model, a unified calculation step can be used, so that details are not omitted and the results convergence is not difficult [23]. The reconstructed *u*_*ce*_ is applied to BC circuit of CCM operation. Fig 5 shows the equivalent circuit.

The parasitic inductance *L*_*l*_ in joint model includes not only the parasitic inductance in wire, but also the parasitic inductance of IGBT itself. The parasitic inductance in this part has a significant impact on IGBT characteristics. *L*_*d*_ and *L*_*s*_ in the figure represent the internal and external parasitic inductance of device, respectively. *L*_*d*_ is given by the component data table. *L*_*s*_ is a numerical value determined by empirical methods. The equivalent load resistance on circuit’s output side is *R*. Eq (10) is the differential formula for *i*_*T*_ in microscopic devices.


$$\frac{di_{T}}{dt}=\frac{1}{L_{l}}(U_{s}-u_{ce}-Ri_{T})$$ (10)

Compared with the equivalent circuit model of IGBT, it has a high degree of similarity. A joint scale model of Buck PEC is established using WD and RA. By analyzing the short time scale characteristics of PET, the evaluation results of switch devices’ safety domain in PET can be obtained, thereby improving the computational accuracy and efficiency.

### 3.3. Joint scale model and short time scale characteristics analysis of dab converter

**Fig 6:**
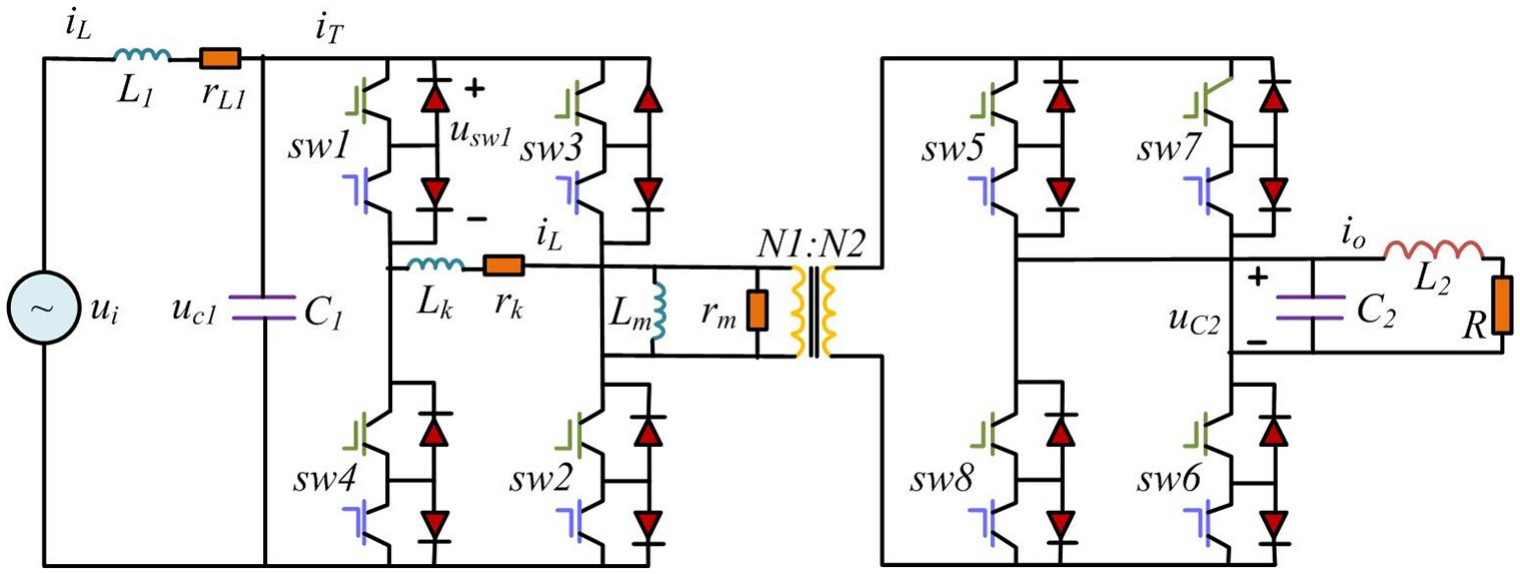
Main circuit topology diagram of dual active bridge power electronic transformer.

Solid State Transformer (SST) is a PEC for AC-AC conversion, which has gradually become a key component in power transmission and management systems [24, 25]. In the distributed energy system of smart grid, Dual Active Bridge-Solid State Transformer (DAB-SST) is used to realize efficient energy conversion between grid and local power generation equipment [26]. By accurately simulating the dynamic response of high-frequency switching processes and magnetic elements, the joint scale model can better predict and manage energy flows, thereby optimizing energy delivery and demand response. DAB converter is a configuration of SST. To verify the application feasibility of joint scale modeling and simulation on such switch converters, DAB-SST is modeled. Fig 6 shows the main circuit topology of transformer.

In the main circuit of DAB-SST, *L*_*k*_ and *r*_*k*_ represent the leakage inductance and internal resistance of high-frequency transformer, respectively. *L*_*m*_ and *r*_*m*_ represent the excitation inductance and internal resistance of high-frequency transformers, respectively. *u*_*i*_ represents the input AC voltage. *L*_1_ and *C*_1_ are the input voltage stabilizing inductance and voltage stabilizing capacitor, respectively. *SW*1~*SW*8 are bidirectional switches. *r*_*L*1_ is the internal resistance of *L*_1_.

*R* is the load resistance. *L*_2_ and *C*_2_ are the output filter inductance and filter capacitor, respectively. *N*_1_ and *N*_2_ are the primary winding turns and the secondary winding turns of high-frequency transformer, respectively. To establish a DAB-SST model, it is necessary to incorporate some coupling relationships between macro-scale and micro-scale models. Therefore, the switch terminal voltage is added to circuit state equation in Eq (11).


$$x={\left(i_{L1}\;i_{Lk}\;i_{Lm}\;i_{o}\;u_{C_{1}}\;u_{C_{2}}\right)}^{T},u=u_{i}-2u_{SW1}$$ (11)

In Eq (11), *i*_*L*1_, *i*_*Lk*_, *i*_*Lm*_, *i*_*o*_, and $u_{C_{1}}$ are the state variables of circuit. *u*_*SW*1_ is the terminal voltage of *SW*1. Eq (12) is the circuit equation for circuit’s state variable.


$$u_{i}=L_{1}\frac{di_{L1}}{dt}+r_{L1}i_{L1}+u_{C1}$$ (12)

In Eq (12), *r*_*L*1_ is the internal resistance of *L*_1_. *u*_*i*_ represents the input alternating current voltage. *L*_1_ is the input voltage stabilizing inductance. Eq (13) represents the circuit equation under modal conditions.


$$L_{m}=\frac{di_{Lm}}{dt}=\frac{N_{1}}{N_{2}}u_{C_{2}}$$ (13)

In Eq (13), *L*_*m*_ represents the excitation inductance of the high-frequency transformer. *N*_1_ and *N*_2_ are the primary winding turns and secondary winding turns of high-frequency transformer, respectively. The DAB-SST state equation can be obtained in Eq (14).


$$\left\{\begin{array}{l} \overset{\cdot }{x}=Ax+Bu\\ x={\left(i_{L1}\;i_{Lk}\;i_{Lm}\;i_{o}\;u_{C_{1}}\;u_{C_{2}}\right)}^{T},u=u_{i} \end{array}\right.$$ (14)

Eq (15) is the terminal voltage expression of switch *SW*1 in the macro-scale circuit model.


$$u_{SW1}(t,1)=\left\{\begin{array}{ll} u_{i} & nT\leq t\leq nT+DT\\ 0 & nT+DT\leq t<(n+1)T \end{array}\right.$$ (15)

In Eq (15), *u*_*SW*1_ is the terminal voltage of *SW*1. *T* is the switching cycle. Eq (16) represents the WD and reconstruction of *u*_*SW*1_ when simulating.


$$u_{SW1}(t)=\sum \overset{\wedge }{h_{1}}(t)u_{V,SW1}(t,1)+\sum \overset{\wedge }{h_{2}}(t)u_{V,SW1}(t,2)$$ (16)

Eq (16) represents the joint scale simulation model of DAB-SST. A simulation model is constructed based on the joint scale model of DAB-SST, with an input voltage of *u*_*i*_ = 120 sin, a switching frequency of 850Hz for each switching device, and a switching period of T = 1.30ms. Table 1 shows the complete simulation parameters.

**Table 1:** Joint scale model construction and simulation model parameters for dual active bridge power electronic transformers.

Parameter symbols	Meaning	Numerical value
Uim	Input AC voltage amplitude	150V
fi	Input AC voltage frequency	70Hz
N1	Number of turns of transformer primary winding	250
N2	Number of turns of transformer secondary winding	150
L1	Input voltage stabilizing inductance	3μH
C1	Input voltage stabilizing capacitor	5μH
RL1	Internal resistance of input voltage stabilizing inductor	5μH
L2	Output rate wave inductance	13μH
C2	Output terminal rate wave capacitance	5μH
N1:N2	Transformer changes	4
f	Switching frequency	850Hz
Lm	Excitation inductance of high-frequency transformer	5mH
Lk	Leakage inductance of high-frequency transformer	10μH
Rk	Internal resistance of high-frequency transformer	1Ω
R	Load resistance	25
D	Duty cycle	0.6
Step	Time step	60ns

The joint scale simulation model can reflect the overshoot phenomenon of terminal voltage and current during each switch cycle when switch is turned off. And its waveform overall presents a sine wave shape. The voltage and current waveforms on the primary side of high-frequency transformer in circuit exhibit positive and negative bidirectional characteristics, with a sine wave trend on both positive and negative half axes. This can reflect how voltage overshoot affects wave on switch tube. The joint scale simulation model can reflect the function of macro-circuit model characteristics and micro-device model characteristics. In this paper, the joint scale model is innovatively proposed by applying WT technology to the modeling of PEC. The new model can more comprehensively and accurately describe the characteristics of PEC in multiple switching states, including linear or nonlinear, dynamic or static characteristics, etc., which is a major breakthrough to the traditional methods. This higher accuracy not only meets the needs of users for safety and electrical characteristics of PEC, but also solves the contradiction of scale problems in simulation calculations. When analyzing the reliability of PEC, it is important to consider fault modes such as overvoltage, overcurrent, and device aging. Using the joint scale model, the converter behavior can be simulated accurately under these extreme operating conditions, potential risks can be identified in advance, and maintenance and protection measures are taken to extend the life of the system.

## 4. Performance analysis of pec joint scale model based on wd and ra

To verify the construction and simulation accuracy of the PEC joint scale model based on WD and RA, a physical experimental circuit of BC running under CCM model is constructed. To ensure the model simulation accuracy, experiments are conducted in the same experimental environment. The IGBT model is KEC KGF40N65KDC. Taking into account the characteristics of the IGBT model used, the test environment should be maintained in the range of 15°C to 35°C as far as possible, and the power supply voltage fluctuations of more than ±5% should be avoided. The modulation strategy of the driving switch is TMS320F28335 digital signal processing. In the experimental circuit, the input DC voltage is 60V, the switching duty cycle is 0.6, the switching frequency is 20kHz, the filtering inductance is 2mH, the filtering capacitance is 2μF, and the load resistance is 30 Ω. In this study, limiting factors and potential sources of error in the experimental environment include, but are not limited to, ambient temperature fluctuations, supply voltage stability, measurement equipment accuracy, and non-ideal characteristics of the switching device itself. Among them, Table 2 shows the peak values of IGBT terminal voltage under different calculation steps.

**Table 2:** Peak voltage of IGBT terminal under different calculation steps.

step = 10-9s	step = 10-8s	step = 2×10-8s	step = 5×10-8s	step = 8×10-8s	step = 10-7s	step = 10-6s
uce						
97.12V	97.23V	97.36V	91.61V	82.76V	91.07V	60.15V
Error rate	0.11%	0.11%	7.76%	15.47%	7.09%	38.43%
u´ce						
97.12V	97.27V	97.12V	96.11V	96.87V	93.91V	60.18V
Error rate	0.022%	0.085%	1.86%	0.91%	4.13%	38.46%

According to Table 2, through the analysis of IGBT terminal voltage peak under different calculation steps, it can be observed that the simulation error decreases with the reduction of calculation steps. This shows that the actual voltage behavior of the simulators can be more accurately simulated on a finer time scale, thus improving the accuracy of the simulation model. However, that a calculation step is too small will greatly increase the simulation time, so it is necessary to balance the simulation accuracy and efficiency in the actual simulation. When using a calculation step size of 10-7s, the peak overshoot voltages of u´_ce_ and u_ce_ were 93.91V and 91.07V, respectively, with an error reduction from 7.09% to 4.13%. When using a calculation step size of 8×10-8s, the peak overshoot voltages of u´_ce_ and u_ce_ were 96.87V and 82.76V, respectively, with an error reduction from 15.47% to 0.91%. When using a calculation step size of 5×10-8s, the peak overshoot voltages of u´_ce_ and u_ce_ were 96.11V and 91.61V, respectively, with an error reduction from 7.76% to 1.86%. When using a calculation step size of 2×10-8s, the peak overshoot voltages of u´_ce_ and u_ce_ are 97.12V and 97.36V, respectively, with an error reduction from 0.11% to 0.085%. When using a calculation step size of 10-8s, the peak overshoot voltages of u´_ce_ and u_ce_ were 97.27V and 97.23V, respectively, with an error reduction from 0.11% to 0.022%. Therefore, using WD and RA, inaccurate simulation results can be corrected in the face of large calculation steps, thereby reducing simulation errors. The results of commonly used methods for calculating step length time for multi-scale problems are compared, as shown in Table 3.

**Table 3:** Comparison of results of commonly used calculation step time methods for multi-scale problems.

Runge-Kutta	Multi-Grid	Finite Element Method	Wavelet Method	Hierarchical multi-scale method	Runge-Kutta
Macro-scale processes require calculation of step time/s					
4.56s	6.77s	5.67s	3.12s	3.68s	4.56s
Micro-scale processes require calculation of step time/s					
2.43s	3.51s	4.61s	2.06s	2.39s	2.43s

**Fig 7:**
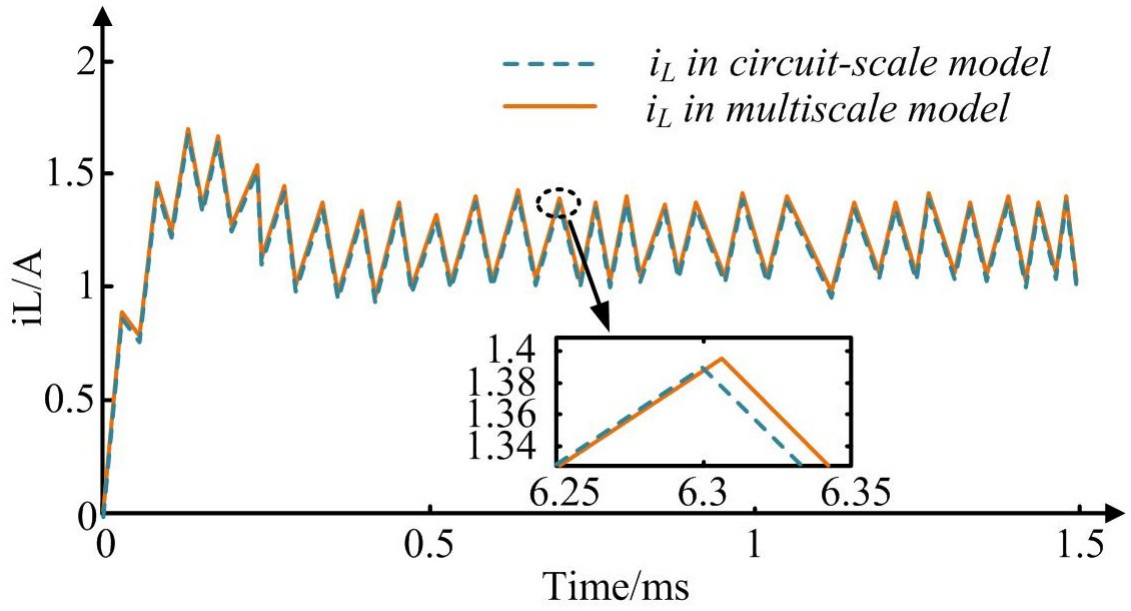
Comparison of inductive current waveforms of buck converters in joint scale model and macro circuit scale model.

As can be seen from Table 3, when dealing with multi-scale problems, the selection and optimization of calculation step length are crucial to ensure accuracy and efficiency. The wavelet method is particularly good in this respect, and the step calculation time required for both macro and micro processes is the lowest. Among them, the calculation step time of the macro process is only 3.12s, while the micro process only needs 2.06s. However, other computational methods perform less satisfactorily on different scales. In the macroscopic process, the calculation step length of the Multi-Grid (MG) method is 6.77s, which is almost twice the time required by the wavelet method. In addition, in the microscopic process, the performance of the Finite Element Method (FEM) is slightly weak, and its calculation step time is as high as 4.61s. Compared with the calculation time of the wavelet method at the same scale, the finite element method needs longer time. It can be seen that the wavelet method is obviously the most superior choice. Other methods have their unique characteristics in some occasions, but they still need to be further optimized in the calculation time. The simulation calculation efficiency of the multi-scale joint simulation model and the single-scale simulation model is compared in Fig 7.

In Fig 7, as simulation duration increases, both the multi-scale joint model and the single-scale model exhibit a waveform state. And the waveform of inductance current *i*_*L*_ in joint simulation model is almost consistent with the waveform in macroscopic circuit scale model when it reaches a stable state. When simulation duration increases to 0.22ms, the inductance current reaches a peak value of 1.68A. When simulation duration increased to 0.59ms, both joint and macro circuit scale simulation models reached a stable state, with an inductance current of around 1.35. Therefore, incorporating the coarse and fine scales of WD and RA into multi-scale joint model can achieve obtaining finer scale information at coarser simulation scales. For different calculation steps, Table 4 shows the time required for joint model and macro circuit scale model to operate for 30 switching cycles.

**Table 4:** Time taken to simulate running 30 switch cycles.

step = 10-9s	step = 10-8s	step = 2×10-8s	step = 5×10-8s	step = 8×10-8s	step = 10-7s	step = 10-6s
Macro circuit scale model						
15.47s	3.81s	3.54s	2.57s	2.63s	2.51s	2.11s
Joint scale model						
16.94s	4.26s	3.73s	3.42s	3.34s	3.13s	2.64s

**Fig 8:**
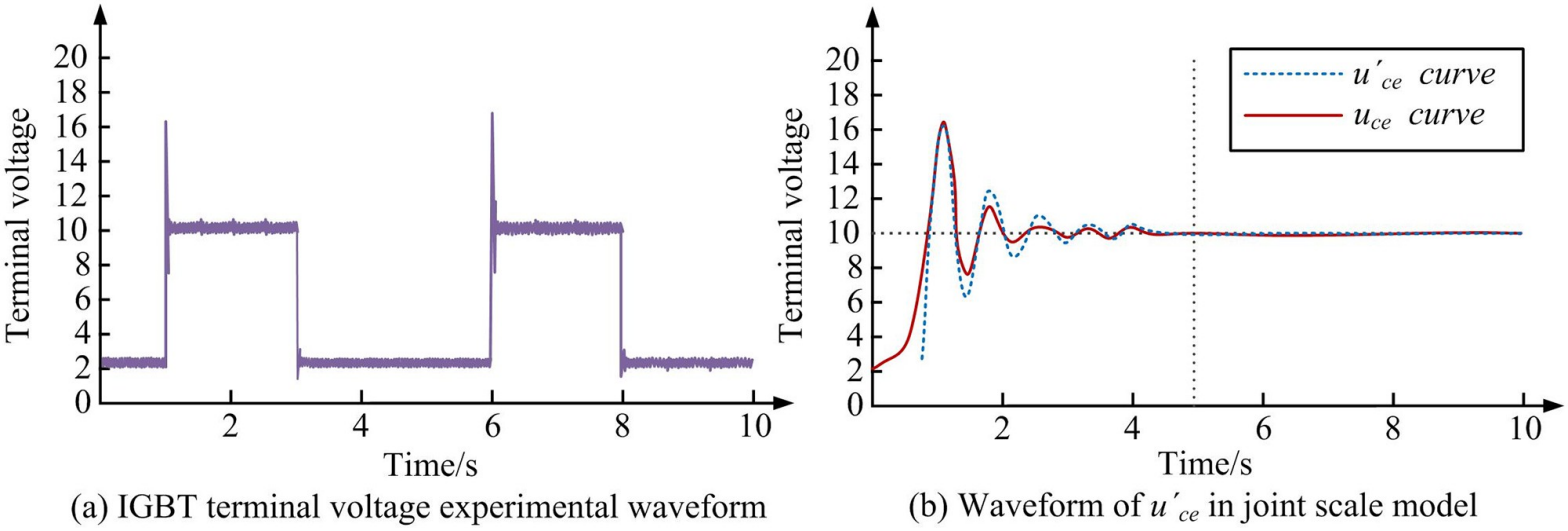
The experimental waveform of IGBT terminal voltage and the waveform of u_’ce_ in the joint scale model. (a) IGBT terminal voltage experimental waveform, (b) Waveform of *u*_*’ce*_ in joint scale model.

According to Table 4, the process of adding coarse and fine scale transformations in joint model does not increase simulation time much and has a small impact on simulation efficiency. In the macro circuit scale model, when the calculation step size is 10^-6^s, the time used is 2.11s. When calculation step size is 10^-7^s, the time used is 2.51s. When calculation step size is 10^-8^s, the time used is 3.81s. When calculation step size is 10^-9^s, the time used is 15.47s. In joint scale model, when using a calculation step of 10^-6^s, the time used is 2.64s. When calculation step size is 10^-7^s, the time used is 3.13s. When using a calculation step of 10-8s, the time used is 4.26s. When using a calculation step of 10^-9^s, the time used is 16.94s. Therefore, when using a suitable calculation step for joint model simulation, this model can not only shorten simulation time, but also reduce simulation errors, increasing computational efficiency and accuracy. In the model verification, the joint scale model with high step size and the macro-circuit scale model with low step size have high consistency, which indicates that WD and RA can help to maintain high simulation accuracy when the computing resources are limited. The joint scale model’s feasibility under WD and RA is analyzed in Fig 8.

**Fig 9:**
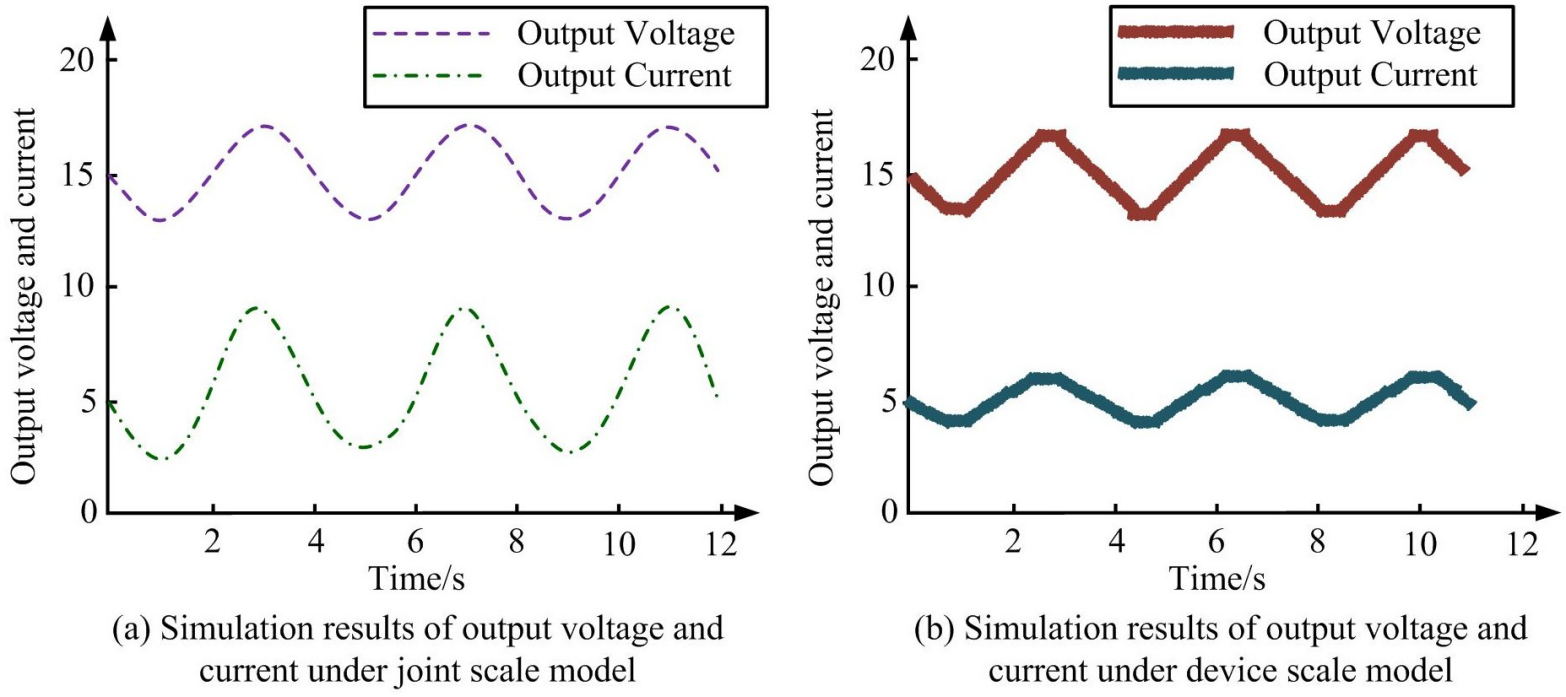
Output voltage and current results under joint scale model and device scale model. (a) Simulation results of output voltage and current under joint scale model, (b) Simulation results of output voltage and current under device scale model.

According to Fig 8, the error between the voltage waveform of control terminal and experimental results is very small, and the overall waveform trend of shutdown delay process is basically consistent. Special attention is paid to the analysis of the sources of experimental errors. They come from the manufacturing tolerances of the IGBT devices, uncertainties in the measurement equipment, small variations in the execution time of the digital signal processor, and the delay of the switch driver circuit. Ignoring these factors that cause changes in disturbance waveform, the peak overshoot voltage of *u*_*ce*_ is 107.5V, and the shutdown delay time is 2 μs. A joint scale model with step = 2×10^-8^s is also used to calculate step size, and a diode internal resistance is added to experimental results for simulation calculations. Therefore, the simulation scale model proposed in this study can present the actual characteristics of BCs. The errors observed in the experiment may come from: measurement errors of the output voltage and current, small deviations in the switching frequency, difference in the algorithm execution time of the signal processor, delay of the drive circuit, etc. The computational performance advantage of WD and RA in simulation mainly comes from its ability to analyze signals at different frequency bandwidths and to analyze the dynamic characteristics of devices in multi-scale time localization. Compared with the traditional Fourier method, WD can capture the behavior change of the switching device in different operating states in real time, optimize the simulation calculation step size, reduce the overall simulation time, and improve the simulation efficiency of the model. Using the same parameters for the joint scale simulation model and device scale model, the simulation results of output voltage and current are obtained, as shown in Fig 9.

**Fig 10:**
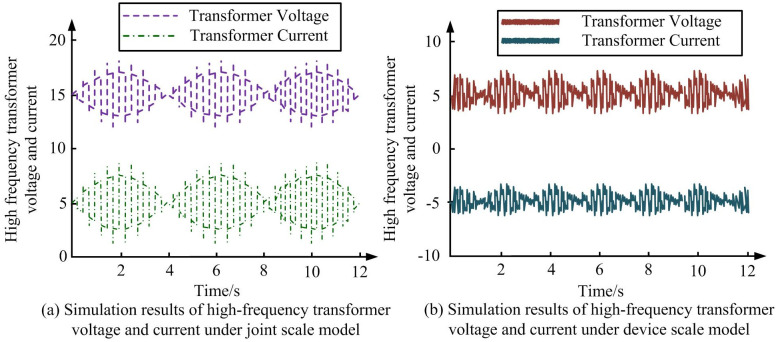
Voltage and current results of bidirectional switching of switching transistor under joint scale model and device scale model. (a) Simulation results of high-frequency transformer voltage and current under joint scale model, (b) Simulation results of high-frequency transformer voltage and current under device scale model.

According to Fig 9, both the output voltage and current of joint simulation model and device scale model generate disturbances, and the error between the two is very small. According to Fig 9(a), the output voltage and current are in a waveform state. The waveform of voltage is more gentle than current, and its peak value is lower than current. According to Fig 9(b), the output voltage and current are in a waveform state. The waveform of voltage is steeper than current, and its peak value is higher than current. Its peak values are also around 1800V and 1.5A, respectively. By observing the graph, the error between the two is relatively small. The waveform state is the graph of inductance current changing with time, which clearly reflects the sinusoidal waveform characteristic of current changing with time. The result of this waveform state means that the joint scale model can accurately capture the periodic current changes of the converter under stable operating conditions. In the power electronics, understanding and predicting the steady-state behavior of circuits are critical, which affect the efficiency and power management of systems. Therefore, the accurate simulation of the output waveform state shows that the combined model can be effectively used to simulate the steady-state and dynamic performance of PEC. The same parameters were still used for joint scale simulation model and device scale model, and the simulation results of high-frequency transformer voltage and current were obtained in Fig 10.

**Fig 11:**
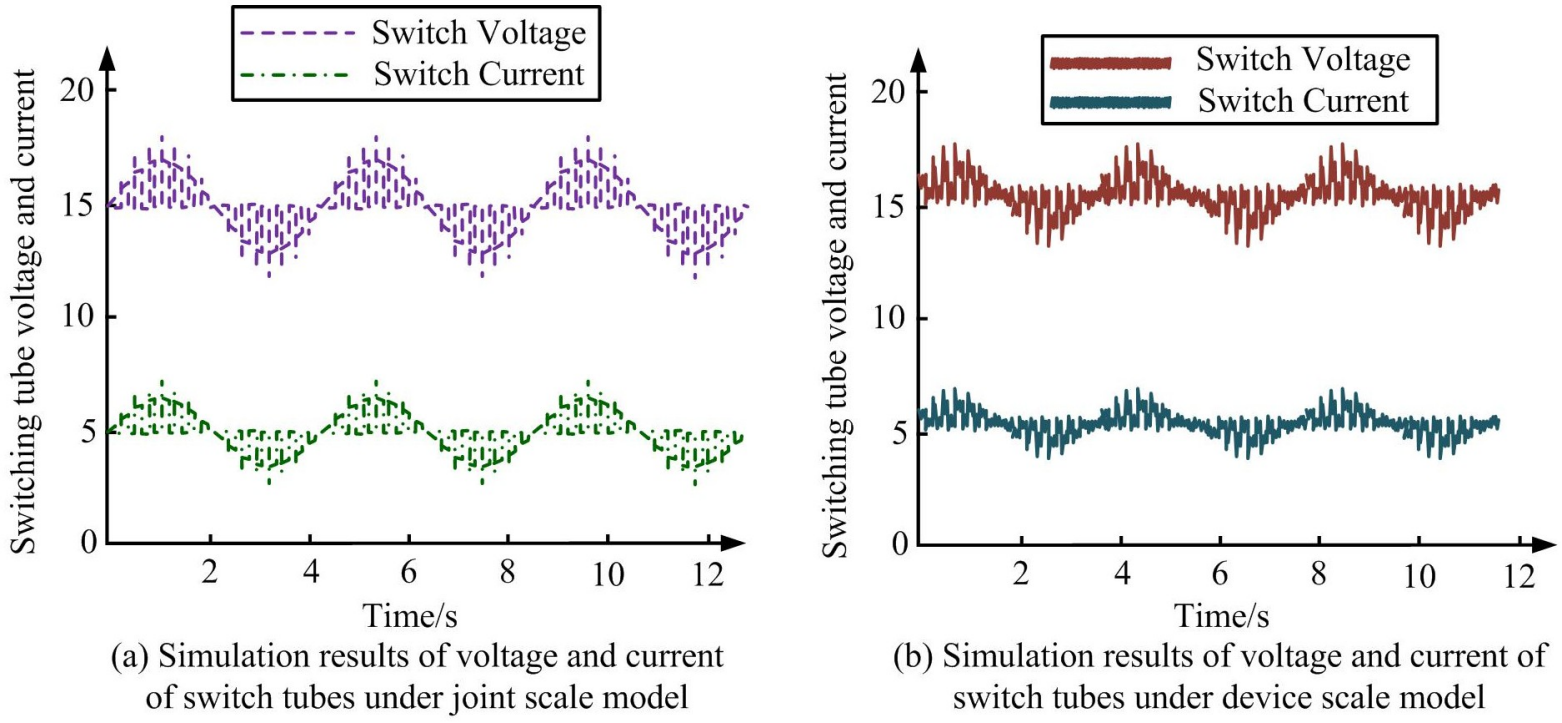
Voltage and current results of bidirectional switching of switching transistor under joint scale model and device scale model. (a) Simulation results of voltage and current of switch tubes under joint scale model, (b) Simulation results of voltage and current of switch tubes under device scale model.

According to Fig 10, the voltage and current of high-frequency transformer in joint simulation model and device scale model showed stable changes. According to Fig 10(a), the voltage value of high-frequency transformer is around 15.3, and the current value is around 5.2. The voltage waveform is narrower than current waveform, and its peak value is lower than current waveform. According to Fig 10(b), the voltage value of high-frequency transformer is around 14.6 and the current value is around -4.8. The waveform of voltage is wider and thicker than current, and its peak value is higher than current. Their peak values are also around 1916V, and through observing graph, the error between simulation and experimental results is less than 4%. Using the same parameters for joint scale simulation model and device scale model, the voltage and current simulation results of switch tube were obtained in Fig 11.

**Fig 12:**
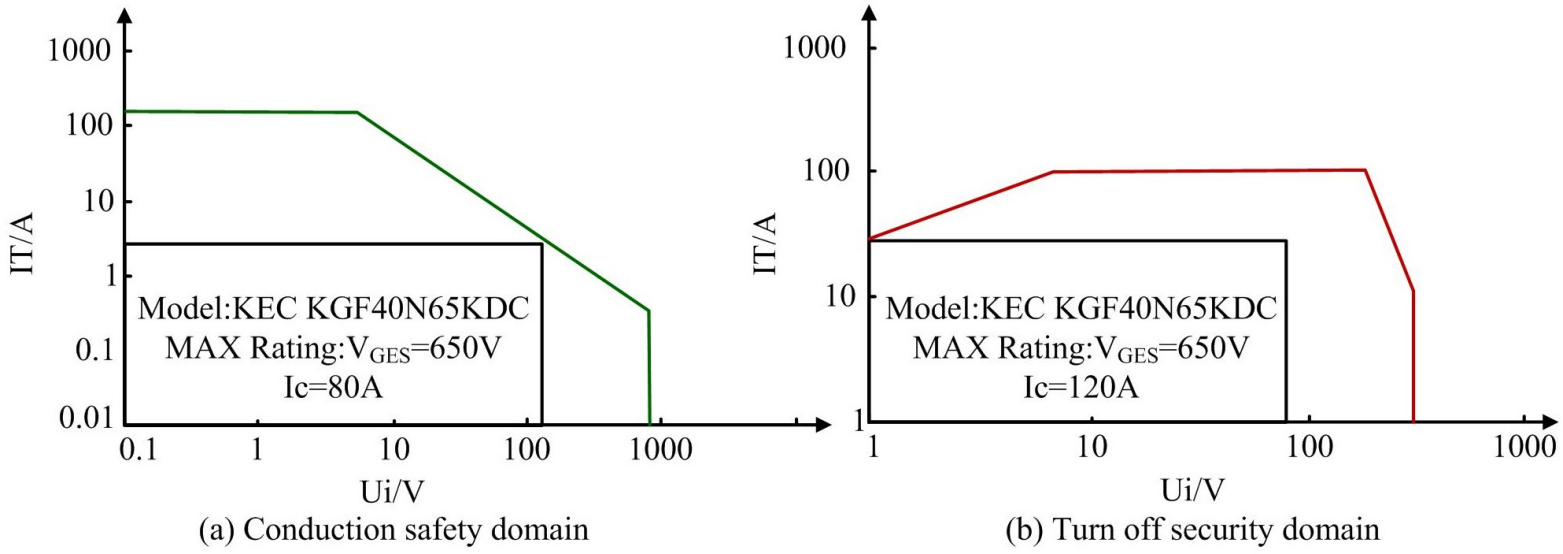
Comprehensive scale model calculation in the simulation process for safe operation and stability assessment. (a) Conduction safety domain, (b) Turn off security domain.

According to Fig 11, the switch voltage and current of joint simulation model and the device scale model exhibit roughly the same variation state. According to Fig 11(a), the voltage value of switch tube is around 15.0, and the current value is around 5.1. The changes in voltage and current show a sinusoidal waveform, with a peak value higher than the current. According to Fig 11(b), the voltage value of switch tube is around 15.2, and the current value is around 5.2. The voltage and current are also sinusoidal waveforms. The peak voltage is higher than the current, with a peak value of around 1916V. The error between simulation and experimental results is less than 5%. As a result, the simulation accuracy of joint scale model is relatively high. The results are roughly the same as in real life, verifying that the multi-scale joint simulation model has certain reference value for the practical application of PEC. The calculation of the comprehensive scale model in the simulation process is evaluated for safe operation and stability, and the results are shown in Fig 12.

According to Fig 12, under certain rated parameters, the joint simulation tests under multiple runs obtained the results of conducting and turning off the device in safe operating range. The evaluation of the conduction safety domain of switching devices is conducted at KEC KGF40N65KDC model, with a maximum rated voltage of 650V and a current of 80A. The evaluation of safety zone for switching devices is based on the model KEC KGF40N65KDC, with a maximum rated voltage of 650V and a current of 120A. According to Fig 12(a), the range of conduction safety zone is at 276A current and 912V voltage. According to Fig 12(b), the range of shutdown safety zone is at 59A current and 596V voltage. The establishment of safety domain is an important index to ensure the normal operation and stability of PEC under harsh working conditions. The safety domain results must take into account the effects of dynamic load changes and network fluctuations encountered by the device in practical applications, which introduce additional uncertainties in the operating environment and may affect the stability and safety of the converter. Therefore, the security domain obtained from simulation testing basically meets the requirements of practical life, further verifying the feasibility of PEC simulation process. The results of safety domain evaluation are critical to the safe operation of PEC. From the evaluation of the on-off and off-off safety domains, the operating boundaries predicted by the simulation model ensure that the device remains within its safe operating range under the extreme conditions. However, in practical applications, the device may encounter more complex load dynamics and network fluctuations, which will affect the safe operation domain of the device. However, the on-off and off-off security domain assessment is still limited by the physical characteristics of the device and environmental factors. For example, the dynamic response due to load fluctuations in practical applications is not fully considered in the simulation model. The Nomenclature table has been summarized into Table 5.

**Table 5:** Nomenclature table.

Abbreviation	Full name
BC	Buck Converter
IHS	Intensity Hue Saturation
SAR	Synthetic Aperture Radar
CCM	Continuous Conduction Mode
IGBT	Insulate-Gate Bipolar Transistor
SST	Solid State Transformer
DAB	Dual Active Bridge
PEC	Power Electronic Converter
WT	Wavelet Transform

## Conclusion

Existing PEC is prone to short-term failure at high frequencies, high loads, and extreme operating conditions, mainly due to device overheating, electrical voltage or current shock damage. Especially in applications with high power switching frequency, such as switching power supplies, electronic control of electric steam death, etc., the thermal stress and electroelectrical stress of the device will be significantly increased, increasing the probability of failure. In addition, the lifetime of switching devices is also limited by their internal structure and material characteristics. These problems are difficult to simulate precisely in the traditional single-scale model, because the physical phenomena and dynamic changes cannot be captured at various scales. PEC is a technology that uses power electronic devices to convert and control electrical energy. Various power converters are widely used in high-voltage direct current transmission, alternating and direct current power transmission, electrolysis, excitation, and other fields. Although existing PECs have played an important role in industrial production, there is still a problem of short-term failure. This study proposes the construction and simulation of a PEC joint scale model based on WD and RA to address the issue of single-scale models and simple combination scale models being unable to accurately describe their characteristics. The process of adding coarse and fine scale transformations in joint model does not increase simulation time much and has a small impact on simulation efficiency. In macro circuit scale model, when calculation step size is 10^-6^s, the time used is 2.11s. When calculation step size is 10^-7^s, the time used is 2.51s. When calculation step size is 10^-8^s, the time used is 3.81s. When calculation step size is 10^-9^s, the time used is 15.47s. In the joint scale model, when calculation step size is 10^-6^s, the time used is 2.64s. When calculation step size is 10^-7^s, the time used is 3.13s. When calculation step size is 10^-8^s, the time used is 4.26s. When calculation step size is 10^-9^s, the time used is 16.94s. Compared with the traditional single-scale model, the joint scale model can reduce two main types of simulation errors: the first type is the time discretization error caused by the large time step. Because it makes the model unable to capture the subtle changes of the device in the fast dynamic process. The second type is the errors caused by spatial discretization, especially in the simulation of the temperature distribution and electromagnetic field distribution of the device. In the joint scale model, the simulation error of the IGBT terminal voltage can be reduced to less than 0.1% at the smallest calculation step (10^-9^s), demonstrating a high degree of accuracy in capturing device behavior. When the joint scale model is used to simulate complex dynamic responses, the time resolution of fine scale can accurately capture the peak voltage and current, thus reducing the error margin. In addition, the spatial discretization errors in the simulation have an important impact on the simulation of the temperature and electromagnetic field distribution of the device. These errors may cause the key parts of the device failure to be ignored, thus affecting the reliability and accuracy of the simulation. The joint scale model can achieve a more accurate description of the internal distribution state of the device by refining the spatial discretization step size and using the waveform fractal feature adaptive method. Therefore, when using a suitable calculation step for joint model simulation, this model can not only shorten simulation time, but also reduce simulation errors, increasing computational efficiency and accuracy. The security domain obtained from simulation testing basically meets the requirements of real life, further verifying the feasibility of PEC simulation process. The construction and simulation of a joint scale model for PEC can accurately describe the physical processes in converter and obtain accurate short-term scale characteristics of converter. Although this study has made progress in improving the accuracy of PEC, it still faces some challenges and has limitations. In practice, the increasing complexity and computational cost of the model may limit its application in large-scale or real-time operating environments. In addition, the precision of the joint scale model strongly depends on the quality of the input parameters and the selection of the wavelet basis function, which requires in-depth understanding of the specific application environment and meticulous parameter adjustment. However, the wavelet used in the simulation of this study is an existing type. For PEC in different application scenarios, such as high-voltage DC transmission and electric drive systems of electric vehicles, the specific operating environment of the equipment may contain other types of changes and disturbances. The complexity of these factors needs further research and exploration of the wavelet.

## Supporting information

S1 Dataset (DOC)

S1 File (DOCX)
