# Supplementary material for: Construction and simulation of a joint scale model for power electronic converters based on wavelet decomposition and reconstruction algorithms
Source: PLoS One. 2024 Apr 5;19(4):e0298590. doi: 10.1371/journal.pone.0298590 (PMC10997115; doi:10.1371/journal.pone.0298590)
Supplement: S1 Dataset — (DOC) [file pone.0298590.s001.doc]

The Data in Figure 7

| iL in circuit-scale model | Time/ms | 0 | 0.5 | 1 | 1.5 |
| --- | --- | --- | --- | --- | --- |
|  | iL/A | 0.02 | 1.41 | 1.26 | 1.13 |
| iL in multiscale model | Time/ms | 0 | 0.5 | 1 | 1.5 |
|  | iL/A | 0.01 | 1.42 | 1.28 | 1.13 |

The Data in Figure 8

| IGBT terminal voltage experimental waveform | | Time/s | 0 | 2 | 4 | 6 | 8 | 10 |
| --- | --- | --- | --- | --- | --- | --- | --- | --- |
|  |  | Terminal voltage | 2.41 | 10.13 | 2.39 | 10.14 | 2.41 | 2.40 |
| Waveform of u´ce in joint scale model | Uce curve | Time/s | 0 | 2 | 4 | 6 | 8 | 10 |
|  |  | Terminal voltage | 2.03 | 9.98 | 10.04 | 9.99 | 10.01 | 10.00 |
|  | u´ce curve | Time/s | 0 | 2 | 4 | 6 | 8 | 10 |
|  |  | Terminal voltage | 2.39 | 9.95 | 10.06 | 10.01 | 10.00 | 10.00 |

The Data in Figure 9

| Simulation results of output voltage and current under joint scale model | | | | | | | | |
| --- | --- | --- | --- | --- | --- | --- | --- | --- |
| Output Voltage | Time/s | 0 | 2 | 4 | 6 | 8 | 10 | 12 |
|  | Output voltage and current | 15.01 | 15.08 | 16.41 | 15.91 | 16.53 | 15.87 | 16.43 |
| Output Current | Time/s | 0 | 2 | 4 | 6 | 8 | 10 | 12 |
|  | Output voltage and current | 5.01 | 5.26 | 5.11 | 4.96 | 5.07 | 5.13 | 5.09 |
| Simulation results of output voltage and current under device scale model | | | | | | | | |
| Output Voltage | Time/s | 0 | 2 | 4 | 6 | 8 | 10 | 12 |
|  | Output voltage and current | 14.99 | 15.12 | 15.98 | 15.96 | 16.05 | 15.98 | 16.06 |
| Output Current | Time/s | 0 | 2 | 4 | 6 | 8 | 10 | 12 |
|  | Output voltage and current | 5.06 | 4.99 | 5.27 | 5.34 | 5.51 | 5.46 | 4.96 |

The Data in Figure 10

| Simulation results of high-frequency transformer voltage and current under joint scale model | | | | | | | |
| --- | --- | --- | --- | --- | --- | --- | --- |
| Transformer Voltage | Time/s | 2 | 4 | 6 | 8 | 10 | 12 |
|  | High frequency transformer voltage and current | 16.98 | 15.06 | 17.13 | 15.04 | 17.09 | 15.03 |
| Transformer Current | Time/s | 2 | 4 | 6 | 8 | 10 | 12 |
|  | High frequency transformer voltage and current | 7.96 | 5.03 | 8.02 | 5.02 | 7.99 | 5.02 |
| Simulation results of high-frequency transformer voltage and current under device scale model | | | | | | | |
| Transformer Voltage | Time/s | 2 | 4 | 6 | 8 | 10 | 12 |
|  | High frequency transformer voltage and current | 5.84 | 5.91 | 5.82 | 6.17 | 5.88 | 5.94 |
| Transformer Current | Time/s | 2 | 4 | 6 | 8 | 10 | 12 |
|  | High frequency transformer voltage and current | -4.29 | -4.36 | -4.28 | -4.05 | -4.31 | -4.28 |

The Data in Figure 11

| Simulation results of voltage and current of switch tubes under joint scale model | | | | | | | |
| --- | --- | --- | --- | --- | --- | --- | --- |
| Switch Voltage | Time/s | 2 | 4 | 6 | 8 | 10 | 12 |
|  | Switching tube voltage and current | 15.06 | 15.07 | 15.12 | 15.01 | 15.09 | 14.76 |
| Switch Current | Time/s | 2 | 4 | 6 | 8 | 10 | 12 |
|  | Switching tube voltage and current | 5.02 | 4.99 | 5.13 | 5.11 | 5.36 | 4.78 |
| Simulation results of voltage and current of switch tubes under device scale model | | | | | | | |
| Switch Voltage | Time/s | 2 | 4 | 6 | 8 | 10 | 12 |
|  | Switching tube voltage and current | 14.67 | 15.26 | 14.84 | 16.53 | 14.37 | 15.14 |
| Switch Current | Time/s | 2 | 4 | 6 | 8 | 10 | 12 |
|  | Switching tube voltage and current | 5.08 | 5.81 | 4.98 | 5.43 | 5.01 | 5.71 |

The Data in Figure 12

| MAX Rating:VGES=650V Ic=80A | | | | | | |
| --- | --- | --- | --- | --- | --- | --- |
|  | Ui/V | 0.1 | 1 | 10 | 100 | 1000 |
| Conduction safety domain | IT/A | 128.19 | 128.19 | 83.07 | 4.62 | 0.01 |
| MAX Rating:VGES=650V Ic=120A | | | | | | |
| Turn off security domain | Ui/V | 1 | 10 | 100 | 1000 | / |
|  | IT/A | 49.86 | 100.05 | 100.05 | 1.00 | / |
