# Supplementary material for: Construction and simulation of a joint scale model for power electronic converters based on wavelet decomposition and reconstruction algorithms
Source: PLoS One. 2024 Apr 5;19(4):e0298590. doi: 10.1371/journal.pone.0298590 (PMC10997115; doi:10.1371/journal.pone.0298590)
Supplement: S1 File — (DOCX) [file pone.0298590.s002.docx]

**Supporting information**

This article designs a joint scale model construction and simulation of power electronic converters based on wavelet decomposition and reconstruction algorithms. This study proposes a joint scale model construction and simulation for power electronic converters based on wavelet decomposition and reconstruction algorithms to address the problem of difficulty in describing the characteristics of single scale models and simple combination scale models. In order to verify the effectiveness of the proposed method, a comparative experiment of the optimization algorithm was designed and the experimental results were analyzed, providing a reference for the optimization of the scale joint model of power electronic converters.

This article mainly studies the construction and simulation of joint scale models for power electronic converters. By applying wavelet decomposition and reconstruction algorithm techniques, more joint scale models have been obtained, providing a reference for the accuracy research of power electronic converters on a short time scale.

Although there have been many achievements in the research on wavelet decomposition and reconstruction algorithms at this stage. However, with the continuous development of science and technology and the increasing demand of people, the current power electronic converters cannot fully meet the needs of modern society on a short time scale. To this end, the research innovatively proposes the construction and simulation of a joint scale model for power electronic converters based on wavelet decomposition and reconstruction algorithms, aiming to effectively improve the physical characteristics and security functions of power electronic converters.
